# High‐dose vitamin D supplementation in patients with COVID‐19: A meta‐analysis of randomized controlled trials

**DOI:** 10.1002/fsn3.3875

**Published:** 2023-12-07

**Authors:** Zhaoshuang Zhong, Long Zhao, Yan Zhao, Shuyue Xia

**Affiliations:** ^1^ Department of Respiratory Central Hospital, Shenyang Medical College Shenyang China

**Keywords:** COVID‐19, high dose, vitamin D

## Abstract

The efficacy of administering high doses of vitamin D to patients diagnosed with COVID‐19 remains uncertain. We conducted a comprehensive search across multiple databases (PubMed, EMBASE, Cochrane Library, and ISI Web of Science) from inception until August 2022, with no limitations on language, to locate randomized controlled trials (RCTs) that investigated the impact of high‐dose vitamin D supplementation (defined as a single dose of ≥100,000 IU or daily dose of ≥10,000 IU reaching a total dose of ≥100,000 IU) on COVID‐19 patients. Risk ratios (RR) with 95% confidence intervals (CI) and weighted mean differences (WMD) with 95% CI were calculated. Our meta‐analysis included 5 RCTs with a total of 834 patients. High‐dose vitamin D supplementation did not show any significant benefits for mortality (*I* ^2^ = 0.0%, *p* = .670; RR 1.092, 95% CI 0.685–1.742, *p* = .711) or intensive care unit (ICU) admission (*I* ^2^ = 0.0%, *p* = .519; RR 0.707, 95% CI 0.454–1.102, *p* = .126) in COVID‐19 patients compared to the control group. However, it was found to be safe and well‐tolerated (*I* ^2^ = 0.0%, *p* = .887; RR 1.218, 95% CI 0.930–1.594, *p* = .151). Subgroup analysis also showed no benefits in overall mortality, including for patients with vitamin D deficiency (*I* ^2^ = 0.0%, *p* = .452; RR 2.441, 95% CI 0.448–13.312, *p* = .303) or compared to the placebo (*I* ^2^ = 0.0%, *p* = .673; RR 1.666, 95% CI 0.711–3.902, *p* = .240). Our research indicates that there is no evidence to support the efficacy of high‐dose vitamin D supplementation in improving clinical outcomes among individuals with COVID‐19, in line with previous studies focused on contexts including rickets. Considering the limitations of the study, additional research may be required.

## Graphical abstract

The effects of high‐dose vitamin D supplementation on patients with COVID‐19 remain unclear. A high dose of vitamin D supplementation demonstrated no benefits for clinical outcomes in patients with COVID‐19.

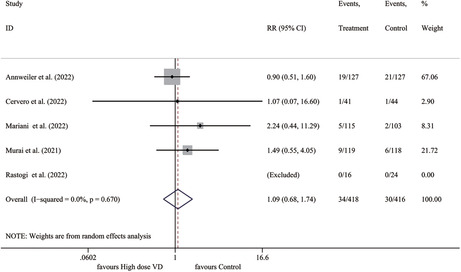

## 1 INTRODUCTION

Coronavirus disease‐2019 (COVID‐19) is caused by the severe acute respiratory syndrome coronavirus 2 (SARS‐CoV‐2), and it has emerged as a global pandemic that has significantly impacted our daily lives. As reported, older adults and individuals with severe underlying medical conditions are at a higher risk of morbidity and mortality from the disease (Wiersinga et al., 2020). Vitamin D, a cholesterol‐derived steroid similar to cortisol, is known to play a role in both innate and acquired immunity (Aglipay et al., 2017; Khammissa et al., 2018), and there has been growing interest in investigating the relationship between vitamin D status and its potential impact on the course and prognosis of COVID‐19 in patients (Mercola et al., 2020).

Several observational studies have reported an association between low levels of 25(OH)D and increased risk and severity of COVID‐19 (Baktash et al., 2021; De Smet et al., 2021; Meltzer et al., 2020; Vassiliou et al., 2020), and vitamin D supplementation, especially in individuals with vitamin D insufficiency and deficiency, may improve survival rates in older adults with COVID‐19 (Alcala‐Diaz et al., 2021; Ling et al., 2020; Nogues et al., 2021). Vitamin D may have protective effects through various mechanisms, such as anti‐inflammatory action to suppress the cytokine storm (Peng et al., 2021; Teymoori‐Rad et al., 2019) and increased expression of the angiotensin‐converting enzyme 2 (ACE2) to alleviate the activation of the renin‐angiotensin system after SARS‐CoV‐2 infection (Coperchini et al., 2022; Getachew & Tizabi, 2021). However, the benefits of vitamin D supplementation for patients with COVID‐19 were mostly supported by observational studies or non‐randomized trials (D'Ecclesiis et al., 2022), and high‐dose vitamin D, particularly intermittent or single high‐dose bolus vitamin D, had been reported with no efficacy in any context, including the prevention of fractures, rickets, and all‐cause mortality (Griffin et al., 2021; Mazess et al., 2021). Therefore, the effectiveness and safety of high‐dose vitamin D supplementation in COVID‐19 patients remain inconclusive, particularly for high‐dose supplementation with potential adverse effects in older adults. To address this issue, we conducted a meta‐analysis of recently published randomized controlled trials (RCTs) to assess the efficacy and safety of high‐dose vitamin D supplementation in COVID‐19 patients.

## 2 METHODS

### 2.1 Literature search

In accordance with the Preferred Reporting Items for Systematic Reviews and Meta‐Analyses (PRISMA) statement guidelines (Page et al., 2021), we conducted a systematic search of the PubMed, EMBASE, Cochrane Library, and ISI Web of Science databases. The search covered the period from inception to August 2022, with no language restrictions, to identify all RCTs that reported on the effects of high‐dose vitamin D supplementation in patients with COVID‐19. Our search utilized various combinations of keywords such as “Vitamin D” or “Cholecalciferol,” “COVID,” “High dose,” and “Randomized.”

### 2.2 Inclusion criteria

We selected RCTs for inclusion in our study if they met the following criteria: (1) they were RCTs; (2) they compared high‐dose vitamin D supplementation (defined as a single dose of ≥100,000 IU or daily dose of ≥10,000 IU reaching a total dose of ≥100,000 IU (Kearns et al., 2014)) with a standard dose or placebo in patients with COVID‐19; and (3) they reported on at least one of the following outcomes of interest: mortality, ICU admission, length of hospital stay, or adverse events. We excluded systematic reviews, narrative reviews, conference papers, case studies, and overlapping trials from the analysis.

### 2.3 Data extraction and quality assessment

Two researchers, Z.‐S. Z. and L.Z., independently reviewed each citation for eligibility based on the title, abstract, and full text. Data from eligible studies were extracted and recorded using a pre‐designed structured data abstraction form. The data collected included baseline characteristics of the participants, such as the author's name, publication year, sample size, mean age, coexisting medical conditions, blood biochemistry data, and length of follow‐up, as well as outcomes such as mortality, admission to the ICU, length of hospital stay, and adverse events. The Modified Jadad scale (Oremus et al., 2001) was used to evaluate the methodological quality of each trial, and any discrepancies in study selection or data extraction between the two researchers were resolved by consensus or referred to a third investigator, S.‐Y.X.

### 2.4 Statistical analysis

The statistical analysis was conducted using STATA version 12.0 with the metan function. For binary outcome data, the results were presented as risk ratios (RR) with 95% confidence intervals (CI), while for continuous data, the weighted mean difference (WMD) with 95% CI was used. Heterogeneity was assessed using *I* (Aglipay et al., 2017) statistics, and a random‐effects (RE) model was applied regardless of the level of heterogeneity, following the recommendation (Cumpston et al., 2019). When significant heterogeneity was found across studies, sensitivity or subgroup analysis was performed. Begg's test was used to assess the funnel plot asymmetry, but only when there were at least ten studies included in the analysis (Begg & Mazumdar, 1994). The level of statistical significance was set at *p* < .05.

## 3 RESULTS

### 3.1 Selected studies and baseline characteristics

**FIGURE 1:**
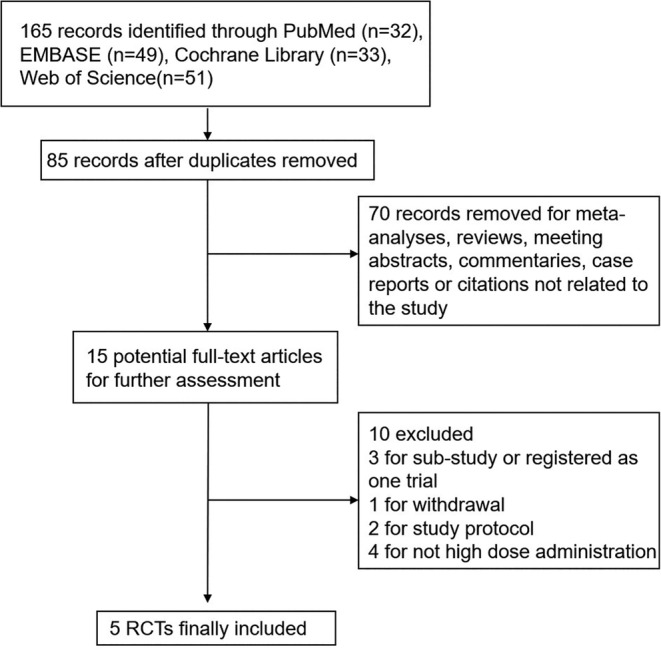
Flowchart of study selection.

We identified a total of 165 citations through our search strategy, from which 15 articles were selected for full‐text assessment after removing duplicates, reviews, case reports, meeting abstracts, or irrelevant papers. Of the 15 articles, we excluded 10 for various reasons, including being sub‐studies or analyses of previous studies, withdrawn by the journal (Lakkireddy et al., 2021), study protocols, or not meeting our high‐dose definition (Caballero‐Garcia et al., 2021; Entrenas Castillo et al., 2020; Karonova, Chernikova, et al., 2022; Maghbooli et al., 2021). Finally, the search strategy leaves us with five RCTs involving 834 patients for our meta‐analysis (Annweiler et al., 2022; Cervero et al., 2022; Mariani et al., 2022; Murai et al., 2021; Rastogi et al., 2022). The flow of the study is depicted in Figure 1, and Table 1 presents the baseline characteristics of the eligible trials.

**TABLE 1:** Characteristics of included studies.

Study	Year	HG/CG	Sample size, *n*	Age, years	Males, *n* (%)	BMI, kg/m^2^	Hypertension, *n* (%)	Diabetes, *n* (%)	25(OH)D, ng/mL	Calcium, mg/dL
Annweiler et al. (2022)	2022	HG	127	87 (81–92)^a^	61 (48)	NR	86 (68)	32 (25)	21.2 (10.4–33.7)^a^	9.0 (8.6–9.2)^a^
		CG	127	89 (83–93)^a^	45 (35)		91 (72)	20 (16)	17.2 (10.4–26.8)^a^	9.0 (8.7–9.2)^a^
Cervero et al. (2022)	2022	HG	41	67 (58–75)^a^	30 (73)	29.7 (4.3)^b^	23 (56)	11 (27)	15.3 (6.3)^b^	8.7 (0.5)^b^
		CG	44	64 (44–72)^a^	30 (68)	30.6 (4.8)^b^	18 (41)	8 (18)	14.3 (6.2)^b^	8.7 (0.4)^b^
Mariani et al. (2022)	2022	HG	115	59.8 (10.7)^b^	64 (55.7)	28.4 (25.8–32.8)^a^	47 (40.9)	32 (27.8)	32.5 (27.2–44.2)^a^	8.8 (8.5–9.0)^a^
		CG	103	58.3 (10.6)^b^	51 (49.5)	27.7 (25.6–32.6)^a^	47 (45.6)	26 (25.2)	30.5 (22.5–36.2)^a^	8.7 (8.5–8.9)^a^
Rastogi et al. (2022).	2022	HG	16	50 (36–51)^a^	6 (37.5)	NR	NR	NR	8.6 (7.1–13.1)^a^	9.4 (9.2–9.7)^a^
		CG	24	47.5 (39.3–49.2)^a^	14 (58.3)				9.54 (8.1–12.5)^a^	8.8 (8.0–9.2)^a^
Murai et al. (2021)	2021	HG	119	56.5 (13.8)^b^	70 (58.8)	31.9 (6.5)^b^	67 (56.3)	49 (41.2)	21.2 (10.1)^b^	8.7 (0.5)^b^
		CG	118	56.0 (15.0)^b^	63 (53.4)	31.4 (7.6)^b^	58 (49.2)	35 (29.7)	20.6 (8.1)^b^	8.7 (0.5)^b^

Study	Year	HG/CG	Time from symptoms onset to admission or enrollment, days	Study location	Follow‐up
Annweiler et al. (2022)	2022	HG	3 (2–4)^a^ ^,^ ^c^	Hospital and nursing home	28 days
		CG	3 (2–4)^a^ ^,^ ^c^		
Cervero et al. (2022)	2022	HG	7 (6–10)^a^ ^,^ ^d^	Hospital	14 days
		CG	7 (6–10)^a^ ^,^ ^d^		
Mariani et al. (2022)	2022	HG	7 (5–10)^a^ ^,^ ^d^	Hospital	30 days
		CG	8 (5.5–10)^a^ ^,^ ^d^		
Rastogi et al. (2022)	2022	HG	NR	Hospital	14 days
		CG			
Murai et al. (2021)	2021	HG	10.2 (3.9)^b^ ^,^ ^c^	Hospital	41 days
		CG	10.4 (4.7)^b^ ^,^ ^c^		

Abbreviations: 25(OH)D, 25‐hydroxyvitamin Vitamin D; BMI, body mass index; CG, control group; HG, high dose vitamin D group; NR, not reported.

^a^ Median interquartile range.

^b^ Mean (SD).

^c^ From onset to enrollment.

^d^ From onset to admission.

### 3.2 Quality assessment and publication bias

We assessed the methodological quality of the included studies using the Modified Jadad scale (Oremus et al., 2001), which evaluated factors such as randomization, blinding methods, rates of withdrawals and dropouts, and allocation concealment. The results are shown in Table 2, with scores ranging from 4 to 7. As the meta‐analysis only included five RCTs, a funnel plot and Begg's test were not performed to assess the risk of publication bias.

**TABLE 2:** Assessment of methodological quality of included studies (Oremus et al., 2001).

Author	Randomization	Double blinding	Allocation concealment	Withdrawals/dropouts	Scores
Annweiler et al. (2022)	Yes	Open‐label	Unclear	Yes	4
Cervero et al. (2022)	Yes	Single‐blind	Unclear	Yes	4
Mariani et al. (2022)	Yes	Yes	Yes	Yes	7
Rastogi et al. (2022)	Yes (method unclear)	Unclear	Unclear	Yes	4
Murai et al. (2021)	Yes	Yes	Yes	Yes	7

### 3.3 Meta‐analysis results

#### 3.3.1 Mortality

**FIGURE 2:**
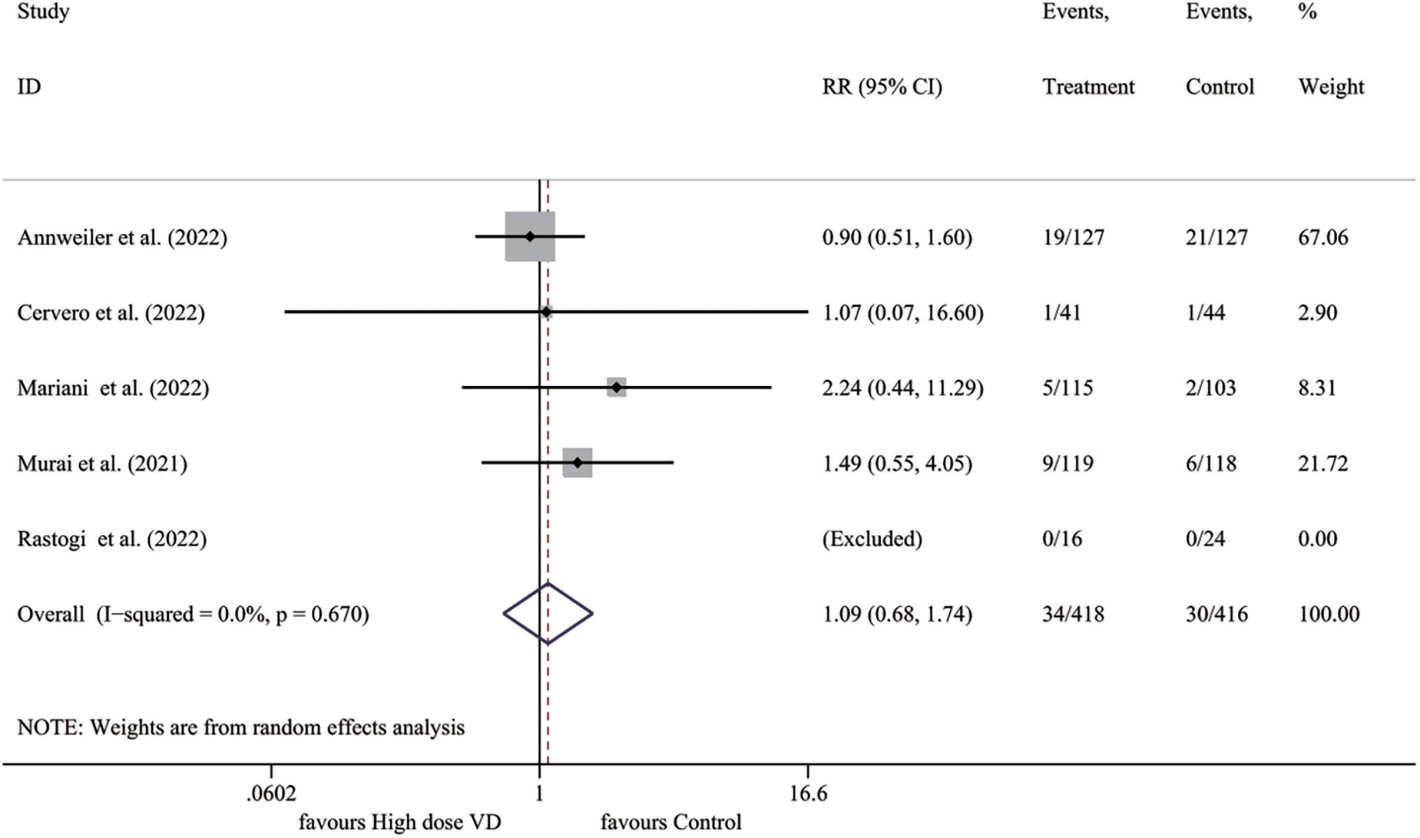
Forest plot for mortality. RR, relative risk; VD, vitamin D.

We included all five RCTs in the meta‐analysis to assess the incidence of death and found that the mortality rate was 8.13% (34/418) in the high‐dose vitamin D group and 7.2% (30/416) in the control group. However, our analysis did not find a significant improvement in mortality with high‐dose vitamin D supplementation compared to the control group (*I* ^2^ = 0.0%, *p* = .670; RR 1.092, 95% CI 0.685–1.742, *p* = .711), as shown in Figure 2.

#### 3.3.2 ICU admission

**FIGURE 3:**
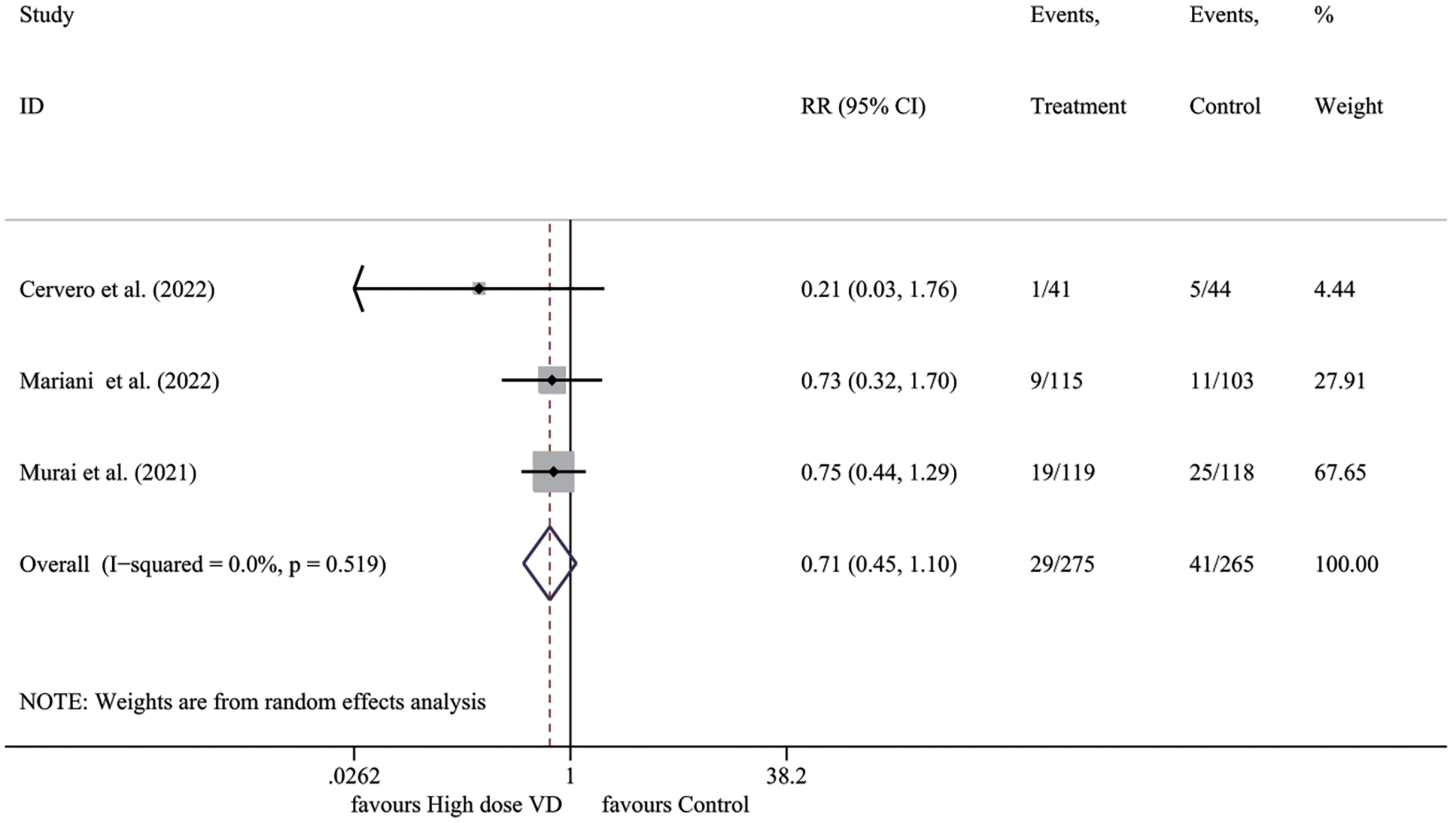
Forest plot for ICU admission rate. ICU, intensive care unit; RR, relative risk; VD, vitamin D.

The rate of ICU admission was reported in three trials, including a total of 540 participants. The results revealed that the use of high‐dose vitamin D did not lead to a significant reduction in the rate of ICU admission (*I* ^2^ = 0.0%, *p* = .519; RR 0.707, 95% CI 0.454–1.102, *p* = .126) (Figure 3).

#### 3.3.3 Length of hospital stay

Data on the length of hospital stay was reported in three RCTs, but due to the use of different data presentation methods, such as median with interquartile range (IQR), a meta‐analysis could not be performed. Nonetheless, the findings from all three studies were consistent, showing no significant difference in the length of hospital stay between the vitamin D group and the control group.

#### 3.3.4 Adverse events

**FIGURE 4:**
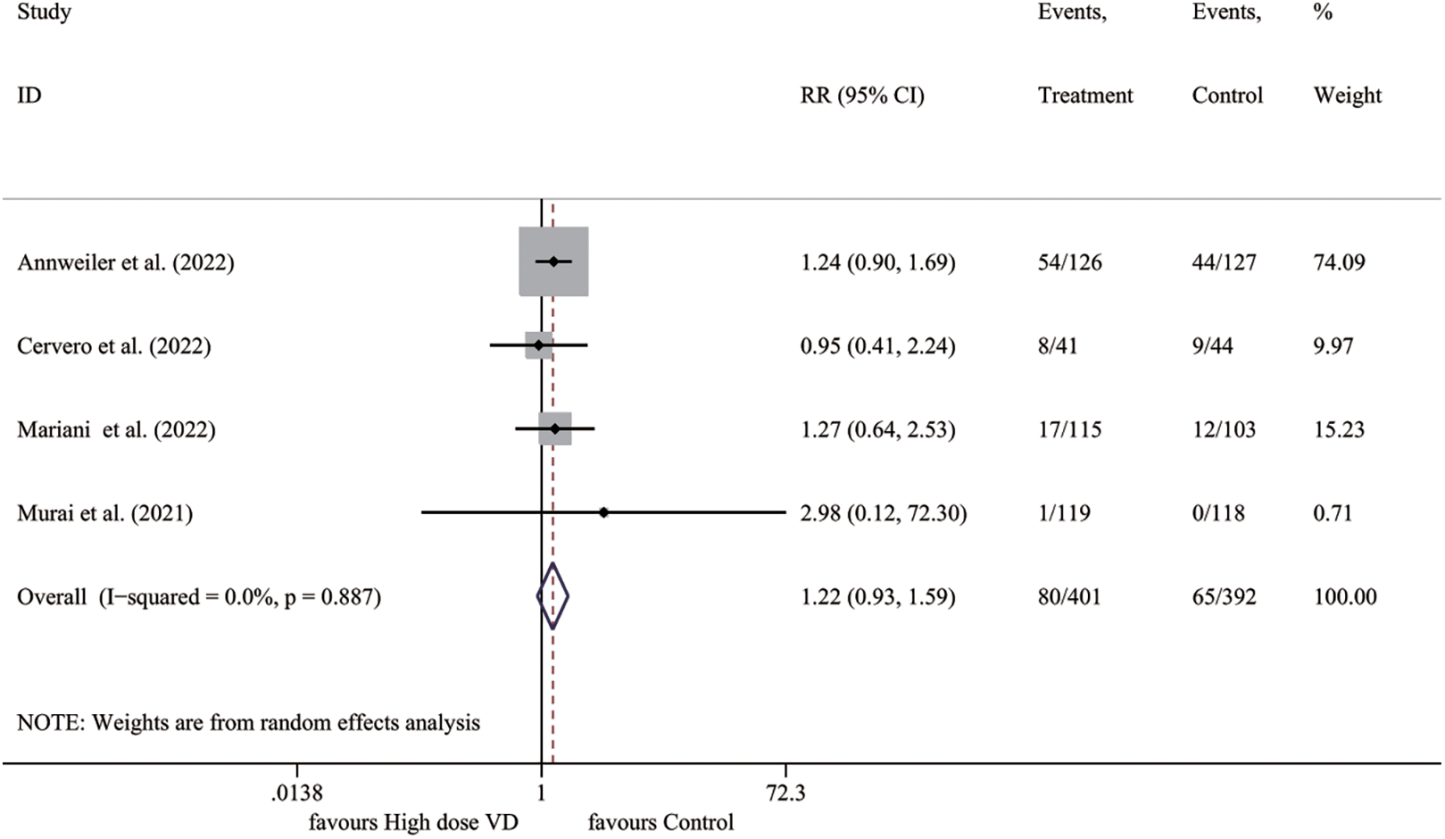
Forest plot for adverse events rate. RR, relative risk; VD, vitamin D.

We collected data on the incidence of adverse events from four studies, but most did not provide a clear association with vitamin D supplementation. The meta‐analysis revealed no significant difference between the two groups in terms of adverse events (*I* ^2^ = 0.0%, *p* = .887; RR 1.218, 95% CI 0.930–1.594, *p* = .151) (Figure 4).

#### 3.3.5 Vitamin D deficiency population

**FIGURE 5:**
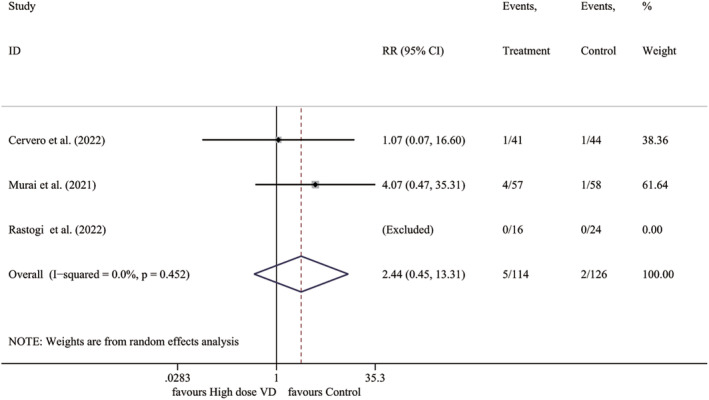
Forest plot for mortality in patients with vitamin D deficiency. RR, relative risk; VD, vitamin D.

Data from three RCTs conducted with participants who had vitamin D deficiency or a subgroup with vitamin D deficiency was analyzed. The findings suggested that there was no significant difference in mortality between the high‐dose vitamin D group and the control group (*I* ^2^ = 0.0%, *p* = .452; RR 2.441, 95% CI 0.448–13.312, *p* = .303) (Figure 5).

#### 3.3.6 High‐dose vitamin D versus placebo

**FIGURE 6:**
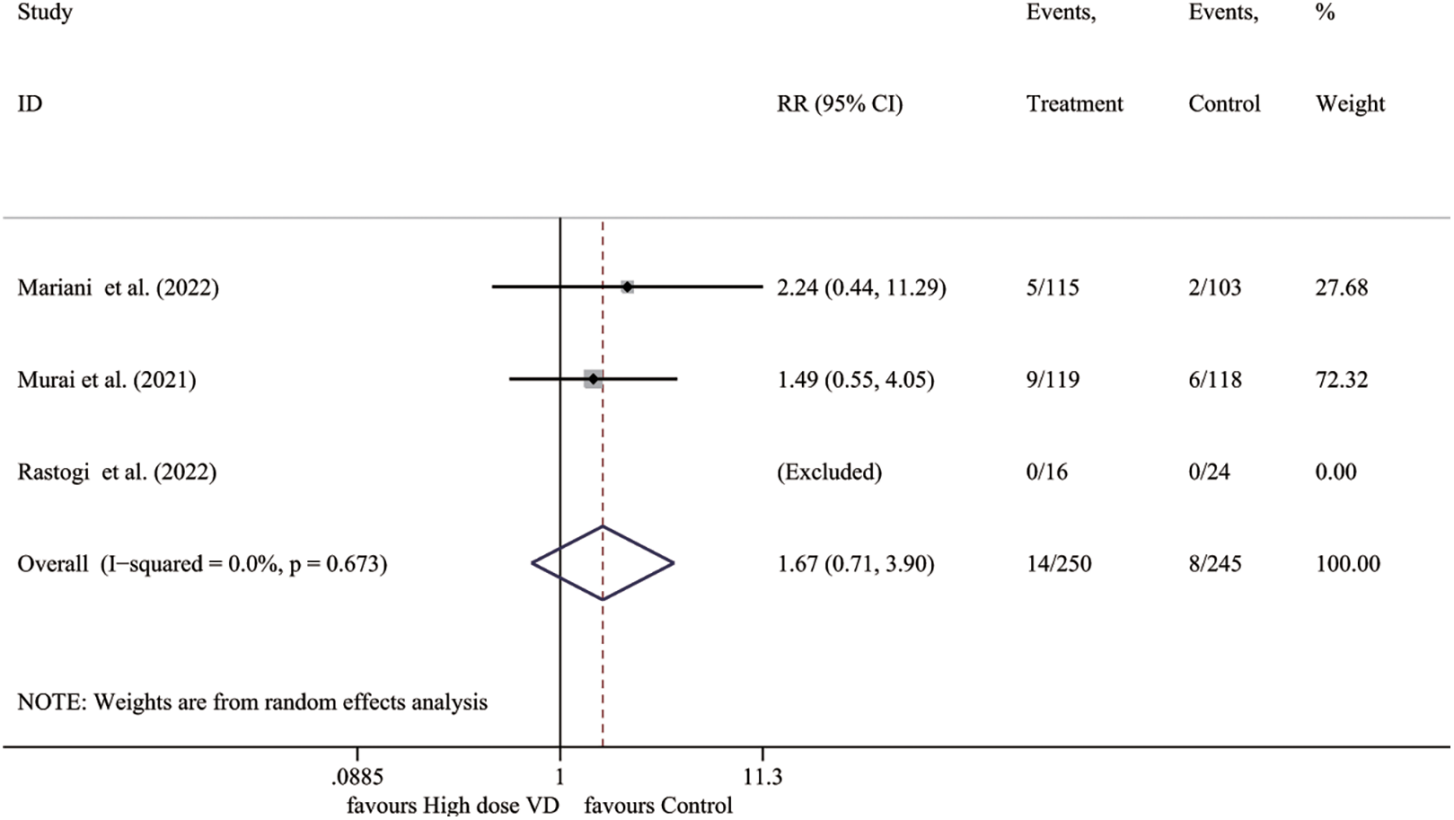
Forest plot for mortality compared to placebo. RR, relative risk; VD, vitamin D.

Three studies compared the efficacy of high‐dose vitamin D to that of a placebo. The findings revealed that there was no significant improvement in mortality rates associated with high‐dose vitamin D supplementation when compared to the placebo group (*I* ^2^ = 0.0%, *p* = .673; RR 1.666, 95% CI 0.711–3.902, *p* = .240) (Figure 6).

## 4 DISCUSSION

Through our meta‐analysis of RCTs, we discovered that administering high‐dose vitamin D to patients with COVID‐19 did not yield a notable improvement in mortality or ICU admission rates compared to the control group. Our subgroup analyses also revealed no significant benefits in overall mortality, whether among patients with vitamin D deficiency or compared to the placebo group. Despite the absence of significant adverse events, our findings suggest that high‐dose vitamin D supplementation may not be effective in enhancing clinical outcomes for individuals with COVID‐19.

Vitamin D, a cholesterol‐derived steroid similar to cortisol, is known to play a role in regulating the immune system and inflammatory response (Bilezikian et al., 2020), which could be important in the prognosis of COVID‐19 patients (Oristrell et al., 2022). Additionally, vitamin D can raise the expression of ACE2 (Coperchini et al., 2022; Getachew & Tizabi, 2021). Although SARS‐CoV‐2 uses ACE2 as a cellular entry receptor (Tai et al., 2020), the upregulation of ACE2 may help balance the ACE/ACE2 ratio and prevent overactivation of the renin‐angiotensin‐aldosterone system (Murray et al., 2020). Studies have shown a positive correlation between vitamin D levels and clinical outcomes in respiratory infections, especially in populations with lower 25‐hydroxyvitamin D levels (Jolliffe et al., 2021; Pham et al., 2021). Several studies have also observed this relationship in COVID‐19 patients (Carpagnano et al., 2021; Petrelli et al., 2021). According to research, people with lower levels of vitamin D are more susceptible to SARS‐CoV‐2 infection and are likely to experience more severe symptoms and higher mortality rates (Karonova, Kudryavtsev, et al., 2022; Rhodes et al., 2021). For instance, a study conducted in the United States found that COVID‐19 patients with a 25(OH)D level of 15 ng/mL had a 20% higher risk of hospitalization and a 53% higher risk of mortality than those with a 25(OH)D level of 40 ng/mL (Seal et al., 2022). However, the majority of these studies were observational, and the results were not conclusive.

Recently, a number of RCTs have been conducted in an effort to establish whether vitamin D supplementation can help to enhance the prognosis of COVID‐19. However, several of these trials employed higher doses of vitamin D for various reasons. On the one hand, a high dose of vitamin D might lead to more rapid increases in plasma 25(OH)D levels, thereby potentially offering quicker protective benefits (Kearns et al., 2014). On the other hand, it is widely acknowledged that administering daily or weekly physiologic doses of vitamin D can lead to enhanced clinical outcomes (Martineau et al., 2017), and some studies were even conducted without a placebo group due to ethical considerations (Annweiler et al., 2022). However, using a higher dose of vitamin D instead of the standard dose can potentially lead to a classification of a “drug” rather than a supplement, raising concerns about uncertain therapeutic effects and potential adverse events.

In previous analyses, Hosseini et al. found that vitamin D could significantly reduce the mortality and ICU admission rates of COVID‐19 patients, but the “regimens without bolus doses appeared to have stronger preventive effects against both COVID‐19 mortality and ICU admission rate compared to bolus doses” (Hosseini et al., 2022); Zhang et al. (2023) concluded that vitamin D did not have a significant impact on reducing mortality and ICU admission among COVID‐19 patients, which denied the benefits of physiological vitamin D as well. All these investigations were not focused on the effects of high‐dose vitamin D supplementation. Actually, research did show that a high dose of vitamin D increases plasma 25(OH)D levels differently than physiologic doses (Bandeira et al., 2022) and may not result in an improved immune response or better clinical outcomes compared to standard, chronic supplementation (Annweiler et al., 2021). Jolliffe et al. (2021). discovered that only regular daily dosing of 400–1000 IU/day effectively prevented clinically evident respiratory infections, while higher dosing or bolus doses administered weekly or monthly did not show the same protective effects. Griffin et al. (2021) and Mazess et al. (2021). also reported that high‐dose vitamin D, particularly intermittent or single high‐dose bolus vitamin D, had no efficacy in any context, including the prevention of fractures, rickets, and all‐cause mortality, which may be due to reasons such as the induction of inactivating 24‐hydroxylase and fibroblast growth factor (FGF) 23, which in turn suppresses activating 1‐hydroxylation. Our study, which involved 5 RCTs and 834 patients, specifically focused on high‐dose vitamin D supplementation, resulting in more precise conclusions. It also provides a new perspective for explaining the contradictory findings in many previous studies. In conclusion, it is important to reconsider using a higher dose of vitamin D for COVID‐19 patients, even though it has been deemed safe and well‐tolerated thus far.

To the best of our knowledge, this study represents the first meta‐analysis to investigate the impact of high‐dose vitamin D supplementation on patients with COVID‐19. However, certain limitations must be acknowledged. Firstly, due to the inclusion of only five RCTs, the sample size may not have enough statistical power to identify differences between groups for some outcomes, and a funnel plot to assess publication bias was not conducted. Among the studies, only three were placebo‐controlled, and only one provided separate data for patients with vitamin D deficiency, which could impact the quality of our study, as vitamin D might not be expected to demonstrate substantial efficacy in patients who were already replete. Secondly, only two of the trials included in the analysis were double‐blinded, raising concerns about the statistical robustness of the findings. Thirdly, the definition of “high dose” varied among the trials, potentially impacting the results with different biological effects (Martineau et al., 2017). For instance, Murai et al. administered a single oral dose of 200,000 IU, which is unlikely to exhibit efficacy in any context, as previously discussed (Murai et al., 2021). Lastly, the trials included in the study had a wide range of other characteristics, including the potentially subjective and variable criteria for ICU admission, the presence of other medical conditions, the initial levels of plasma 25(OH)D, and the length of the follow‐up period. The time from COVID‐19 symptom onset to admission or enrollment also varied across the studies, potentially rendering the interventions too late to effectively correct any vitamin D‐related deficiency in immune regulation in certain trials. The diversity in these factors could reduce the robustness of the analysis, and it is essential to exercise caution when interpreting the study findings. Although it is challenging to perform a placebo‐controlled trial of vitamin supplementation in individuals likely or known to be vitamin deficient, an ideal study of vitamin D in COVID‐19 would have been an extensive community‐based comparison of prophylactic daily dose (max 1000 IU/day) replacement versus placebo in vitamin D‐deficient individuals, with mortality as the primary endpoint.

## 5 CONCLUSION

Our study's findings revealed that administering high‐dose vitamin D supplements did not yield any noticeable clinical benefits for COVID‐19 patients relative to the control group, regardless of whether they had vitamin D deficiency or were given a placebo. However, given the limitations of the study, additional research is warranted to corroborate these findings.

## AUTHOR CONTRIBUTIONS

**Zhaoshuang Zhong:** Conceptualization (equal); investigation (equal); writing – original draft (lead). **Long Zhao:** Formal analysis (equal); methodology (equal). **Yan Zhao:** Formal analysis (equal); methodology (equal). **Shuyue Xia:** Conceptualization (equal); investigation (equal); writing – review and editing (lead).

## CONFLICT OF INTEREST STATEMENT

The authors affirm that they have no conflicts of interest related to this study.

## Data Availability

The data that support the findings of this study are available on request from the corresponding author.
